# Urban-Rural Differences in Bone Mineral Density: A Cross Sectional Analysis Based on the Hyderabad Indian Migration Study

**DOI:** 10.1371/journal.pone.0140787

**Published:** 2015-10-20

**Authors:** Heli T. Viljakainen, Yoav Ben-Shlomo, Sanjay Kinra, Shah Ebrahim, Hannah Kuper, K. V. Radhakrishna, Bharati Kulkarni, Jon H. Tobias

**Affiliations:** 1 Musculoskeletal Research Unit, School of Clinical Sciences, University of Bristol, Bristol, United Kingdom; 2 Children's Hospital, Helsinki University Central Hospital and University of Helsinki, Helsinki, Finland; 3 School of Social and Community Medicine, University of Bristol, Bristol, United Kingdom; 4 Department of Non Communicable Disease Epidemiology, London School of Hygiene and Tropical Medicine, London, United Kingdom; 5 South Asia Network for Chronic Disease. Public Health Foundation of India, New Delhi, India; 6 Department of Clinical Research, London School of Hygiene & Tropical Medicine, London, United Kingdom; 7 National Institute of Nutrition, Hyderabad, India; Georgia Regents University, UNITED STATES

## Abstract

**Background:**

Fracture risk is rising in countries undergoing rapid rural to urban migration, but whether this reflects an adverse effect of urbanization on intrinsic bone strength, as reflected by bone mineral density (BMD), is currently unknown.

**Methods:**

Lumbar spine (LS) and total hip (TH) BMD, and total body fat and lean mass, were obtained from DXA scans performed in the Hyderabad arm of the Indian Migration Study (54% male, mean age 49 years). Sib-pair comparisons were performed between rural-urban migrants (RUM) and rural non-migrated (RNM) siblings (N = 185 sib-pairs).

**Results:**

In analyses adjusted for height, gender, age and occupation, rural to urban migration was associated with higher lumbar and hip BMD and greater predicted hip strength; ΔLS BMD 0.030 (0.005, 0.055) g/cm^2^, ΔTH BMD 0.044 (0.024; 0.064) g/cm^2^, Δcross-sectional moment of inertia 0.162 (0.036, 0.289) cm^4^. These differences were largely attenuated after adjusting for body composition, insulin levels and current lifestyle factors ie. years of smoking, alcohol consumption and moderate to vigorous physical activity. Further analyses suggested that differences in lean mass, and to a lesser extent fat mass, largely explained the BMD differences which we observed.

**Conclusions:**

Rural to urban migration as an adult is associated with higher BMD and greater predicted hip strength, reflecting associated alterations in body composition. It remains to be seen how differences in BMD between migration groups will translate into fracture risk in becoming years.

## Introduction

Age-specific hip fracture rates are declining in North America [1, 2] and Europe [3–5] but rising in Asia [6, 7]. For example, between 1990–2 and 2002–6, hip fractures rates amongst those over 70 years increased 3.4 fold in Beijing, China [8]. Although the reasons for this increase are unclear, an adverse effect of urbanization on bone health is thought to be responsible. Urbanization is associated with several lifestyle factors which are potentially deleterious for bone including reduced weight bearing physical activity, smoking and alcohol intake, availability of sugary and fatty foods, and less outdoor activities leading to reduced vitamin D stores. Interestingly, in a previous cohort study in Norway, participants living in densely populated areas were found to have a higher prevalence of forearm fractures compared with those living in more sparsely populated areas [9]. Rather than a lack of weight bearing physical activity, which is not expected to affect the upper limb, the latter finding suggests either that urbanization is associated with a generalized deficit in bone mass and strength, or alternatively that urbanization leads to a greater risk of falls.

There have been very few studies examining relationships between urbanization and bone mass per se. In a study comparing bone mineral density (BMD) between rural and urban populations in Thailand, femoral neck BMD was found to be higher in males and females living in rural areas compared with urban dwellers from Bangkok, while little difference was observed at the lumbar spine [10]. The observed difference at the hip but not the lumbar spine is consistent with the hypothesis that reduced weight bearing physical activity makes at least some contribution to the apparent increase in risk of hip fracture following urbanization. However, ecological studies of this nature may be subject to unknown bias and confounding, particularly in this instance given that the rural group comprised of participants from just two villages in a single district.

The sib-pair design, in which rural to urban migrants (RUM) are compared with their rural non-migrant (RNM) siblings, represents a more robust approach to comparing characteristics between rural and urban populations, given this is less susceptible to bias and confounding. Based on this approach, the Hyderabad arm of the Indian Migration Study (HIMS) reported large increases in obesity and type 2 diabetes mellitus (DM) associated with rural to urban migration [11]. Type 2 DM is also associated with adverse effects on bone health, as reflected by an increased risk of hip fractures [12]. However, despite their higher fracture risk, subjects with type 2 DM have a higher lumbar spine and femoral neck BMD [13], which is also seen in association with insulin resistance [14–18]. Conversely, after adjustment for body mass index (BMI), BMD has frequently been found to be reduced in those with metabolic syndrome [14, 16–18], suggesting that if anything, insulin independently may exert a negative influence on BMD, as supported by our recent findings from the Avon Longitudinal Study of Parents and Children [19, 20].

In the present investigation, we exploited the Hyderabad arm of IMS to determine whether rural to urban migration is associated with differences in BMD or other skeletal characteristics related to bone strength. We used this study design to determine (i) whether rural to urban migration is associated with differences in BMD, (ii) if these differences are more marked at the hip as compared with the lumbar spine, possibly indicating a role of altered weight bearing physical activity, (iii) whether differences in BMD between groups are explained by those in body composition or insulin resistance, and (iv) whether any other differences in bone strength are present, as ascertained from hip structural analysis [21].

## Methods

### Study Population

The Indian Migration Study (IMS) was comprised rural to urban migrants and their spouses recruited from four factories in India (Lucknow, Hindustan Aeronautics Ltd; Nagpur, Indorama Synthetics Ltd; Hyderabad, Bharat Heavy Electricals Ltd; and Bangalore, Hindustan Machine Tools Ltd) and their siblings who had remained in a rural area. The original fieldwork for the IMS was conducted between 2005 and 2007, during which time 1995 participants were examined in Hyderabad (overall response rate for IMS 50%) [22]. All 1995 participants of the Hyderabad arm of the IMS were invited to attend a clinic at the National Institute of Nutrition between January 2009 and December 2010. The current analyses were based on the cross-sectional data obtained during these clinical investigations. For the present study only sib-pairs were included (N = 185) in which one sibling had migrated to the city (rural-urban-migrant, RUM) and the other had stayed in a rural area (rural-non-migrant, RNM).

### DXA scanning

Subjects underwent total body, lumbar spine (LS) and hip scans using Hologic scanners (Hologic Discovery A model for 93% of scans; Hologic QDR 4500 Elite machine for 7% of scans). Scanners distributed similarly between migration groups (Chi-Sq: P = 0.115) but Hologic QDR 4500 Elite machine was more frequently used in females compared to males (8.2% vs 4.6%; p = 0.040). Scans were visually inspected, and those with major movement or other artefacts in the region of interest were excluded as incomplete scans. LS scans showing pathological changes such as osteoarthritis affecting a single vertebra were re-analysed after this was excluded; where changes affected two or more vertebrae the LS scan was excluded. Whole body fat and lean mass (expressed in kg) were derived from total body scans. Areal bone mineral density (BMD), expressed as g/cm^2^, was obtained for the LS, total hip (TH) and femoral neck (FN).

Each scan was analyzed using the manufacturer's automated hip structural analysis (HSA) program, which generated a range of structural parameters for the proximal femur at three locations [23]. Parameters describing properties across the narrowest diameter of the femoral neck were used. Geometric indices consisted of neck width (width, cm), neck shaft angle (NSA, degrees), hip axis length (HAL, mm), cross-sectional area (CSA, cm^2^), endocortical diameter (ED, cm), and average cortical thickness (ACT, cm). Derived biomechanical strength indices comprised cross-sectional moment of inertia (CSMI, cm^4^) which reflects structural rigidity, section modulus (Z, cm^3^) which provides a measure of bending resistance, and buckling ration (BR) (= (CSMI / Z) / ACT) which gives an estimate of cortical stability.

### Anthropometric data

We used a digital Seca weighting machine (www.seca.com) to measure weight to the nearest 0.1 kg. We measured standing height to the nearest 1 mm with a plastic stadiometer at the end of expiration (Leicester height measure; Chasmors Ltd, Camden, London, UK). Waist circumference was measured to the nearest mm using a non-stretch narrow metal tape at the narrowest point of the abdomen between the ribs and the iliac crest at the end of expiration. Hip circumference was measured at the widest part of the buttock. Each of these measures was assessed twice, and the average of the two values was used in the analysis.

### Laboratory assessment

Participants were asked to attend fasting and the time of the last meal was recorded. Venous blood samples (20 mL) were collected. Blood samples, were separated and stored at -20°C locally and transported to the All India Institute of Medical Sciences (AIIMS), Delhi. Insulin was assessed through radioimmunoassay. The quality of local assays was checked with regular external standards and internal duplicate assays and monitored by AIIMS.

### Questionnaire data

Participants were interviewed using a structured questionnaire. Type of occupation was recorded into five categories: 0, housework; 1, unemployed; 2, unskilled or semiskilled manual; 3, skilled manual; and 4, non-manual [24]. Smoking was assessed as positive if individuals actively smoked or chewed tobacco. Few individuals reported past but not active smoking. Thus, a number of smoking years was calculated. Use of alcohol was graded with 1 if never used, 2 if previously used, but not anymore or 3 if currently uses alcohol. IMS-PAQ was used to gather information on participant's habitual physical activity, PA. Activity was recalled over the last one-month within specified domains (occupational, household, hobbies, exercise, sedentary behaviors, travel, discretionary and sleep) as described earlier [25]. Frequency and duration of each activity was collected. Metabolic equivalent unit values (METs) were assigned to each activity. Activities were categorized into sedentary < 1.5 METS; light 1.5 < 3 METS; moderate 3–6 METS; vigorous > 6 METS. As only 3% of the sample reported participation in vigorous activity, moderate and vigorous activity was subsequently regrouped as moderate to vigorous physical activity (MVPA).

### Quality assurance

Training of fieldworkers and screening staff was conducted over a two week period by the co-applicants and a local trained epidemiologist and experienced staff, and repeated at the mid-point of the study. The observers were standardised against each other, and inter-observer variation were assessed at the start and mid-point of the study. A pilot study was conducted over a two week period to assess the recruitment and screening procedures. The anthropometric equipment was calibrated at the start of every clinic. The Cardiac Biochemistry Lab, AIIMS, is part of the UK National External Quality Assessment (www.ukneqas.org.uk) programme for quality assurance of the biochemical assays.

### Statistics

Differences in continuous characteristics between rural non-migrants (RNM) and their rural-urban migrant siblings (RUM) were tested with related samples t-test (N = 185 sib-pairs). A change in each exposure and outcome was calculated (e.g. ΔBMD = BMD_RUM_−BMD_RNM_). Difference between groups in bone outcomes were tested with linear regression first without adjustments (crude), or additionally adjusted for height (including difference in sex, height, age, occupation type (dichotomized into non-manual (0), manual work (1)) and finally for differences in all lifestyles and body composition measures (including years of smoking, alcohol consumption and MVPA, lean mass, fat mass and insulin concentrations). Interactions between sex of migrant and bone outcomes were explored by deriving gender specific z-scores for each bone outcomes. Subsequently, ΔBMD z-scores as mentioned above were calculated and regressed against sex of migrant. As no interaction was present (p>0.1), both genders were analyzed together. Values are presented with mean and 95% CI, if not indicated otherwise.

### Ethical approval and consent

Ethical approval was obtained from the AIIMS Ethics Committee, and the ethics committees of the National Institute of Nutrition and the London School of Hygiene & Tropical Medicine. Informed written consent was collected from all participants after detailed information has been given of the procedures of the examination. All participants diagnosed with medical conditions were referred for appropriate treatment.

## Results

### Characteristics of Participants

Between January 2009 and December 2010, 1995 IMS participants were invited to attend a clinic at the National Institute of Nutrition. In total, 918 participants from IMS were examined (Response rate 46%). Clinic attendees did not differ in age from non attendees. 849 (92%) participants attending the research clinic underwent DXA scans of the hip and lumbar spine. 37 hip scans were excluded (28 incomplete scans, 9 major artefacts) as were 24 lumbar spine scans (18 incomplete scans, 3 major artifacts, 3 spinal abnormalities). Satisfactory hip and spine scans, along with complete information on covariates, were available in 764 participants who provided the basis for the present study, of these we identified 185 sib-pairs with complete residential records.

The baseline characteristics are presented in Table 1. The groups were similarly split between males and females, but RUM participants were older than RNM (50 vs. 48 years, respectively). Occupation type differed between groups; 35% of RNM were involved in non manual work while the corresponding number in RUM was 17%. As previously reported, BMI in RUM was higher than in RNM, reflecting differences in lean and particularly fat mass [11]. Compared to RNM, waist-to-hip ratio was higher in RUM. Insulin concentration was higher in RUM compared to RNM. In spite of the fact that a lower proportion of RNM were involved in manual work, energy expenditure and MVPA duration were greater in the RNM group compared with RUMs, presumably reflecting other lifestyle differences between rural and urban environments. There was evidence for greater smoking in the RNM group, whereas alcohol consumption and parity were similar between groups.

**Table 1:** Characteristics of matched groups of RNM and RUM (sibs) with mean and SD.

Variables	RNM		RUM		P-value^1^
N	185		185		
Male (%)	57.3		49.7		0.17^§^
Age (y)	48.1	10.2	50.2	6.4	0.001
Urban years (y)	1.1	3.2	31.2	8.0	<0.001
Smoking (y)	4.3	10.3	2.7	9.1	0.09
Non-manual work (%)	35.1		17.3		<0.001^§^
Never used alcohol (%)	65		69		0.46^§^
Energy expedinture (METs/h)	1.60	0.30	1.51	0.16	<0.001
Moderate to vigorous PA time (min/d)	197	181	111	90	<0.001
Parity	2.9	1.3	2.7	0.9	0.28
Height (m)	1.60	0.09	1.59	0.09	0.16
BMI (kg/m^2^)	23.8	4.3	26.5	3.7	<0.001
Waist-to-hip ratio	0.87	0.07	0.90	0.08	<0.001
Lean mass (kg)	43.7	9.0	45.5	8.7	0.04
Fat mass (kg)	17.2	7.1	21.1	6.3	<0.001
Insulin (mU/L)*	6.0	4.4	8.7	8.1	<0.001

RNM, rural non-migrants; RUM, rural-to-urban migrants

PA, physical activity; BMI, body mass index

^1^paired sample t-test

§chi-square test

*log transformed for statistical tests.

#### BMD and hip structural parameters in RNM and RUM sibs According to Migration Status

BMDs and hip structural variables were compared between RUM and RNM (Table 2). There was no interaction between sex of migration and bone outcomes in the analysis, thus genders were analyzed together. In crude analyses, TH and FN BMDs were higher in RUM compared with RNM: the difference was 0.03 g/cm^2^ (3.1–3.7%). In contrast, no difference was observed in LS BMD. RUM tended to have higher ACT at the femoral neck, while the NSA was smaller compared with RNM, but p-values were only suggestive.

**Table 2:** Bone characteristics with mean and 95% CI for groups and comparisons between RNM and RUM.

	RNM^1^			RUM^2^			P-value^3^
LS BMD, g/cm^2^	0.887	0.862,	0.911	0.901	0.883,	0.920	0.28
TH BMD, g/cm^2^	0.886	0.867,	0.905	0.913	0.895,	0.930	0.02
FN BMD, g/cm^2^	0.727	0.709,	0.746	0.754	0.738,	0.770	0.01
Width, cm	3.18	3.12,	3.24	3.21	3.14,	3.28	0.46
Hip Axis Length, mm	102.5	101,	105	102.0	100,	103	0.62
Cross-sectional Area, cm^2^	2.77	2.69,	2.85	2.85	2.78,	2.91	0.11
Endocortical Diameter, cm	2.82	2.76,	2.89	2.84	2.77,	2.92	0.65
Average Cortical Thickness, cm	0.179	0.174,	0.184	0.184	0.179,	0.190	0.10
Cross-sectional Moment of Inertia, cm^4^	2.33	2.22,	2.44	2.39	2.26,	2.51	0.42
Section Modulus, cm^3^	1.32	1.27,	1.38	1.34	1.29,	1.39	0.61
Buckling Ratio	10.5	10,	11	10.5	10,	11	0.99
Neck Shaft Angle,°	130.0	129,	131	129.2	128,	130	0.08

LS, lumbar spine; TH, total hip; FN, femoral neck

^1^N = 185

^2^N = 185

^3^paired sample t-test.

**Fig 1:**
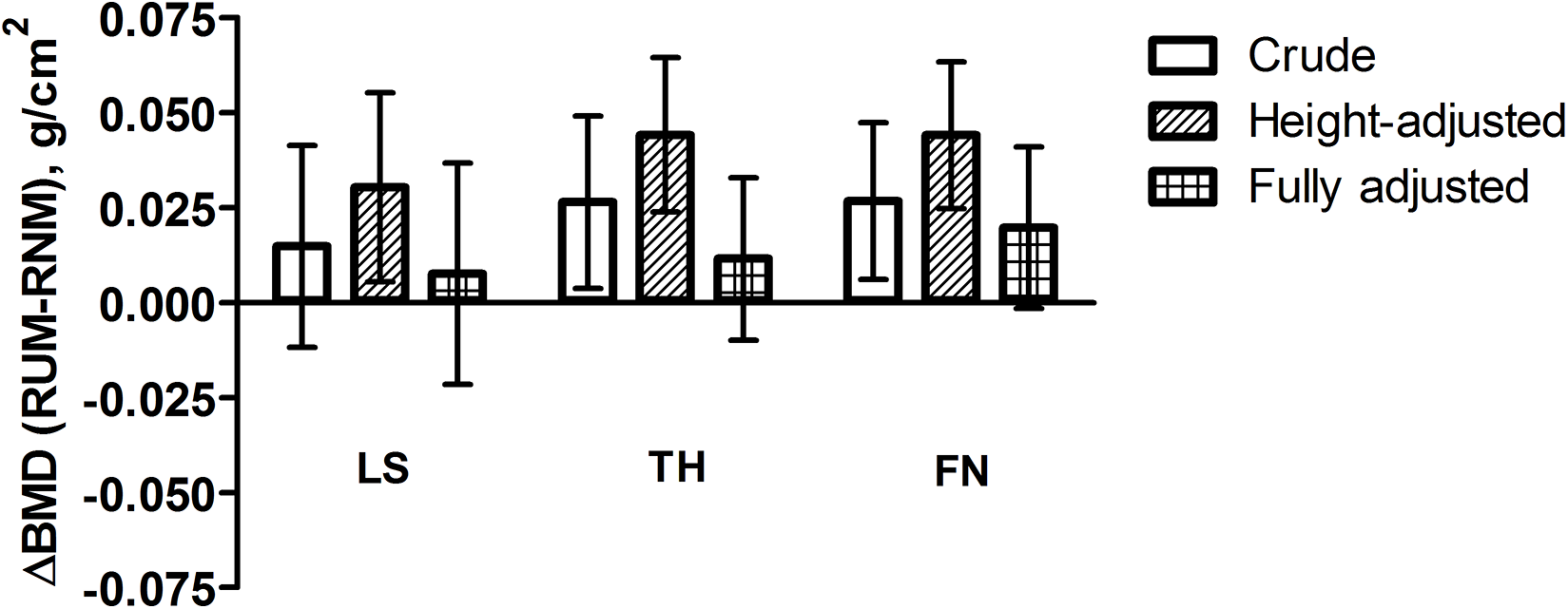
Difference between RUM and RNM in bone mineral density at lumbar spine (LS), total hip (TH), femoral neck (FN). Bars represent mean values with 95% CI for different models: crude, height, and fully adjusted.

After adjusting for differences in sex, height, age, type of occupation (= height-adjusted model), a greater LS BMD was now observed in RUM compared to RNM, and BMD differences at other sites appeared to strengthen (Table 3, Fig 1). In addition, several differences in FN structural parameters were now evident: CSA, ACT, CSMI and Z were greater in RUM compared with RNM. These differences were all attenuated after further adjustment for differences in lifestyle factors and body composition.

**Table 3:** Difference in BMDs and hip structural parameters between RUM and RNM with mean and 95% CI in the three models.

	Mean	95% CI	P-value^1^
**LS BMD, g/cm** ^**2**^			
Crude	0.015	-0.012, 0.041	0.28
Height	0.03	0.005, 0.055	0.02
All	0.008	-0.022, 0.037	0.61
**THIP BMD, g/cm** ^**2**^			
Crude	0.026	0.004, 0.049	0.02
Height	0.044	0.024, 0.064	<0.001
All	0.012	-0.010, 0.033	0.29
**FN BMD, g/cm** ^**2**^			
Crude	0.027	0.006, 0.047	0.01
Height	0.044	0.025, 0.063	<0.001
All	0.020	-0.002, 0.041	0.07
**Width, cm**			
Crude	0.032	-0.053, 0.12	0.46
Height	0.040	-0.047, 0.13	0.37
All	-0.02	-0.123, 0.079	0.67
**Hip Axis Length, mm**			
Crude	-0.634	-3.16, 1.89	0.62
Height	0.202	-1.71, 2.12	0.84
All	-0.227	-2.47, 2.01	0.84
**Cross-sectional Area, cm** ^2^			
Crude	0.075	-0.017, 0.167	0.11
Height	0.156	0.081, 0.230	<0.001
All	0.046	-0.033, 0.124	0.25
**Endocortical Diameter, cm**			
Crude	0.021	-0.070, 0.113	0.65
Height	0.019	-0.076, 0.115	0.69
All	-0.035	-0.147, 0.077	0.54
**Average Cortical Thickness, cm**			
Crude	0.005	-0.001, 0.012	0.10
Height	0.010	0.004, 0.016	0.002
All	0.006	-0.001, 0.014	0.11
**Cross-sectional Moment of Inertia, cm** ^4^			
Crude	0.065	-0.093, 0.223	0.42
Height	0.162	0.036, 0.289	0.01
All	0.011	-0.130, 0.152	0.88
**Section Modulus, cm** ^3^			
Crude	0.018	-0.051, 0.087	0.61
Height	0.072	0.023, 0.122	0.005
All	0.011	-0.045, 0.066	0.70
**Buckling Ratio**			
Crude	0.007	-0.79, 0.81	0.99
Height	-0.343	-1.19, 0.50	0.42
All	-0.282	-1.29, 0.73	0.58
**Neck Shaft Angle,**°			
Crude	-0.814	-1.74, 0.109	0.08
Height	-0.715	-1.70, 0.271	0.15
All	-0.638	-1.80, 0.525	0.28

^1^linear trend

Crude; non adjusted, Height; adjusted for sex, height, age, and occupation type, All; additionally adjusted for smoking, alcohol consumption, MVPA, lean mass, fat mass and insulin.

Associations between confounders/mediators and bone outcomes were tested (S1 Table). To summarize these; age was mostly inversely and LM positively associated with bone outcomes, while effect of FM depended on the outcome. Role of lifestyles was rather small.

## Discussion

To our knowledge, the present study is the first to utilize a sib-pair design to examine associations between rural to urban migration and bone strength. Based on our height adjusted model, we found that rural to urban migration is associated with higher lumbar and hip BMD, and more favorable femoral neck structure resulting in greater estimated hip strength. These differences were entirely explained by associated differences in body composition and lifestyle factors between RNM and RUM, for which differences in fat and particularly lean mass were largely responsible.

Urban migration is associated with numerous lifestyle changes including diet, smoking, alcohol and decreased physical activity. In the present study these were considered as modifiers since migration took place on average at the age of 18 (SD 4.8) years. In addition, we assumed the lifestyles prior to migration were similar to their RNM siblings in childhood and youth. Although differences in lifestyle factors between RUM and RNM did not appear to explain the BMD differences which we found, these were presumably responsible for the major differences in body composition thought to underlie the greater BMD in RUM participants.

Whereas urban migration has previously been reported to be associated with rapid gains in fat mass [11], lean mass also appeared to be increased as presented here. Although differences in lean mass between RUM and RNM were approximately 50% those in fat mass, the former appeared to explain a greater proportion of the difference in BMD, reflecting the considerably stronger relationship between BMD and lean mass as compared with fat mass [26, 27]. Higher lean mass after urban migration may have resulted from higher caloric and/or protein intake. Conversely, in rural villages a prudent diet, typically low in protein, combined with higher energy expenditure may not support muscle growth [28]. Unfortunately were not able to assess the dietary patterns in the present study.

Alternatively, since insulin exerts a trophic effect on muscle tissue, mediated at least partially by the actions of insulin-like growth factor [29], any tendency for urban migration to increase lean mass may have been secondary to higher insulin levels. The latter possibility would imply that increased lean mass contributes to higher BMD in other hyperinsulinemic states, such as the metabolic syndrome. Whereas higher BMD in patients with metabolic syndrome have been found to be attenuated after adjustment for BMI [14, 16–18], since BMI is also related to lean mass, adjustment for lean mass may have resulted in equivalent attenuations in BMD if these measures had been available.

Although BMD is inversely related to fracture risk, the greater BMD in the RUM group was largely in proportion to their greater weight. Therefore, it is difficult to predict the impact of these BMD differences on fracture risk, which was not analysed as part of this study. Similarly, whereas hip structural analysis indicated that RUM participants had larger femoral necks leading to greater predicted strength as reflected by CSMI, this is likely to be in-keeping with the greater loading as a result of their higher weight. Our results are contradictory to some earlier ones [10], but urban-rural differences in BMD seem to vary by affluence and country [30].

Interestingly, the decreased energy expenditure and MVPA in the RUM group did not dilute the effects we observed on bone strength. MVPA does not evaluate rare high impacts which may underlie the relationship between physical activity and BMD [31], and it may be that fewer differences existed between RUM and RNM in terms of the type of physical activity likely to affect BMD. However, our findings of equivalent BMD differences at the lumbar spine and hip also go against a major direct influence of weight bearing physical activity differences between groups on BMD, which would be expected to lead to greater differences at the hip compared to the lumbar spine.

One of the strengths of the present study was the use of sibling pairs to improve the matching between RUM and RNM groups. A further strength was the use of DXA-derived measures of body composition; lean mass and fat mass allowed us to adjust BMD for body composition more accurately than conventional methods based on BMI. The main limitation of this study was its cross sectional design, which makes it difficult to infer causality. Since this is a factory based study, apparent differences which we observed could have been explained by those in work environment. Moreover, our findings cannot be generalized to other migration studies based on different designs. Notwithstanding these reservations, we feel that differences in occupation are unlikely to have contributed to our results, since the differences we observed were unaffected by adjustment for type of occupation Moreover, since rural to urban migrants were self-selected, characteristics of migrants may have differed in important ways prior to migration. However, no differences in height were observed between groups, and analysis of BMD in relation to urban years suggests that in the first few years after migration, BMD values were similar irrespective of group (data not shown). A further weakness is a lack of validated fracture data, and so it remains to be seen how differences in BMD between migration groups will translate into fracture risk.

In conclusion, we compared lumbar spine and hip BMD and structural parameters between siblings in RNM and RUM participants from the Hyderabad arm of the Indian Migration Study. Rural to urban migration was associated with higher lumbar and hip BMD and more favorable hip structure, which appeared to be explained by changes in body composition which are well recognized in this context.

## Supporting Information

S1 Table Bone outcomes and exposures in sibpair design (N = 185) with beta coefficient, SEM and p-value. (DOCX)

## References

[1] Brauer CA, Coca-Perraillon M, Cutler DM, Rosen AB. Incidence and mortality of hip fractures in the United States. JAMA. 2009;302: 1573–1579. 10.1001/jama.2009.1462 19826027 PMC4410861

[2] Leslie WD, O'Donnell S, Jean S, Lagace C, Walsh P, Bancej C, et al Trends in hip fracture rates in Canada. JAMA. 2009;302: 883–889. 10.1001/jama.2009.1231 19706862

[3] Lofman O, Berglund K, Larsson L, Toss G (2002) Changes in hip fracture epidemiology: redistribution between ages, genders and fracture types. Osteoporos Int 13:18–25 11878451 10.1007/s198-002-8333-x

[4] Kannus P, Niemi S, Parkkari J, Palvanen M, Vuori I, Jarvinen M (2006) Nationwide decline in incidence of hip fracture. J Bone Miner Res 21:1836–8 10.1359/jbmr.060815 17002578

[5] Abrahamsen B, Brixen K (2009) Mapping the prescriptiome to fractures in men—a national analysis of prescription history and fracture risk. Osteoporos Int 20:585–97 10.1007/s00198-008-0711-2 18690484

[6] Hagino H, Nakamura T, Fujiwara S, Oeki M, Okano T, Teshima R. Sequential change in quality of life for patients with incident clinical fractures: a prospective study. Osteoporos Int. 2009;20: 695–702. 10.1007/s00198-008-0761-5 18836672

[7] Rowe SM, Song EK, Kim JS, Lee JY, Park YB, Bae BH, et al Rising incidence of hip fracture in Gwangju City and Chonnam Province, Korea. J Korean Med Sci. 2005;20: 655–658 16100460 10.3346/jkms.2005.20.4.655 PMC2782164

[8] Xia WB, He SL, Xu L, Liu AM, Jiang Y, Li M, et al Rapidly increasing rates of hip fracture in Beijing, China. J Bone Miner Res. 2012;27: 125–129. 10.1002/jbmr.519 21956596

[9] Sogaard AJ, Gustad TK, Bjertness E, Tell GS, Schei B, Emaus N, et al Urban-rural differences in distal forearm fractures: Cohort Norway. Osteoporos Int. 2007;18: 1063–1072. 10.1007/s00198-007-0353-9 17333447

[10] Pongchaiyakul C, Nguyen TV, Kosulwat V, Rojroongwasinkul N, Charoenkiatkul S, Rajatanavin R. Effect of urbanization on bone mineral density: a Thai epidemiological study. BMC Musculoskelet Disord. 2005;6: 5 10.1186/1471-2474-6-5 15693996 PMC549192

[11] Kinra S, Andersen E, Ben-Shlomo Y, Bowen L, Lyngdoh T, Prabhakaran D, et al Association between urban life-years and cardiometabolic risk: the Indian migration study. Am J Epidemiol. 2011;174: 154–164. 10.1093/aje/kwr053 21622949 PMC3132275

[12] Janghorbani M, Van Dam RM, Willett WC, Hu FB. Systematic review of type 1 and type 2 diabetes mellitus and risk of fracture. Am J Epidemiol. 2007;166: 495–505. 10.1093/aje/kwm106 17575306

[13] de L II, van der Klift M, de Laet CE, van Daele PL, Hofman A, Pols HA. Bone mineral density and fracture risk in type-2 diabetes mellitus: the Rotterdam Study. Osteoporos Int. 2005;16: 1713–1720. 10.1007/s00198-005-1909-1 15940395

[14] Hwang DK, Choi HJ. The relationship between low bone mass and metabolic syndrome in Korean women. Osteoporos Int. 2010;21: 425–431. 10.1007/s00198-009-0990-2 19565174

[15] Hernandez JL, Olmos JM, Pariente E, Martinez J, Valero C, Garcia-Velasco P, et al Metabolic syndrome and bone metabolism: the Camargo Cohort study. Menopause. 2010;17: 955–961. 10.1097/gme.0b013e3181e39a15 20613668

[16] von Muhlen D, Safii S, Jassal SK, Svartberg J, Barrett-Connor E. Associations between the metabolic syndrome and bone health in older men and women: the Rancho Bernardo Study. Osteoporos Int. 2007;18: 1337–1344. 10.1007/s00198-007-0385-1 17492393

[17] Kim HY, Choe JW, Kim HK, Bae SJ, Kim BJ, Lee SH, et al Negative association between metabolic syndrome and bone mineral density in Koreans, especially in men. Calcif Tissue Int. 2010;86: 350–358. 10.1007/s00223-010-9347-2 20354685

[18] Szulc P, Varennes A, Delmas PD, Goudable J, Chapurlat R. Men with metabolic syndrome have lower bone mineral density but lower fracture risk—the MINOS study. J Bone Miner Res. 2010;25: 1446–1454. 10.1002/jbmr.13 20200928

[19] Lawlor DA, Sattar N, Sayers A, Tobias JH. The association of fasting insulin, glucose, and lipids with bone mass in adolescents: findings from a cross-sectional study. J Clin Endocrinol Metab. 2012;97: 2068–2076. 10.1210/jc.2011-2721 22492875 PMC3387416

[20] Sayers A, Lawlor DA, Sattar N, Tobias JH. The association between insulin levels and cortical bone: findings from a cross-sectional analysis of pQCT parameters in adolescents. J Bone Miner Res. 2012;27: 610–618. 10.1002/jbmr.1467 22095452 PMC3378703

[21] Kaptoge S, Beck TJ, Reeve J, Stone KL, Hillier TA, Cauley JA, et al Prediction of incident hip fracture risk by femur geometry variables measured by hip structural analysis in the study of osteoporotic fractures. J Bone Miner Res. 2008;23: 1892–1904. 10.1359/jbmr.080802 18684092 PMC2686919

[22] Ebrahim S, Kinra S, Bowen L, Andersen E, Ben-Shlomo Y, Lyngdoh T, et al The effect of rural-to-urban migration on obesity and diabetes in India: a cross-sectional study. PLoS Med. 2010;7: e1000268 10.1371/journal.pmed.1000268 20436961 PMC2860494

[23] Beck TJ. Hip Structural Analysis (HSA) Program, (BMD and Structural Geometry Methodology). 2002;Octobert 29: 1–10.

[24] Kinra S, Radha Krishna KV, Kuper H, Rameshwar Sarma KV, Prabhakaran P, Gupta V, et al Cohort profile: Andhra Pradesh Children and Parents Study (APCAPS). Int J Epidemiol. 2014;43: 1417–1424. 10.1093/ije/dyt128 24019421 PMC4190511

[25] Sullivan R, Kinra S, Ekelund U, Bharathi AV, Vaz M, Kurpad A, et al Socio-demographic patterning of physical activity across migrant groups in India: results from the Indian Migration Study. PLoS One. 2011;6: e24898 10.1371/journal.pone.0024898 22022366 PMC3194815

[26] Clark EM, Ness AR, Tobias JH. Adipose tissue stimulates bone growth in prepubertal children. J Clin Endocrinol Metab. 2006;91: 2534–2541. doi: jc.2006-0332 [pii]. 16621904 10.1210/jc.2006-0332 PMC2742729

[27] Gjesdal CG, Halse JI, Eide GE, Brun JG, Tell GS. Impact of lean mass and fat mass on bone mineral density: The Hordaland Health Study. Maturitas. 2008;59: 191–200. 10.1016/j.maturitas.2007.11.002 18221845

[28] Bansal D, Satija A, Khandpur N, Bowen L, Kinra S, Prabhakaran D, et al Effects of migration on food consumption patterns in a sample of Indian factory workers and their families. Public Health Nutr. 2010;13: 1982–1989. 10.1017/S1368980010001254 20507672

[29] Otto A, Patel K. Signalling and the control of skeletal muscle size. Exp Cell Res. 2010;316: 3059–3066. 10.1016/j.yexcr.2010.04.009 20406633

[30] Matsuzaki M, Pant R, Kulkarni B, Kinra S. Comparison of Bone Mineral Density between Urban and Rural Areas: Systematic Review and Meta-Analysis. PLoS One. 2015;10: e0132239 10.1371/journal.pone.0132239 26162093 PMC4498744

[31] Tobias JH, Steer CD, Mattocks CG, Riddoch C, Ness AR. Habitual levels of physical activity influence bone mass in 11-year-old children from the United Kingdom: findings from a large population-based cohort. J Bone Miner Res. 2007;22: 101–109. 10.1359/jbmr.060913 17014381 PMC2742715

