# Supplementary material for: Urban-Rural Differences in Bone Mineral Density: A Cross Sectional Analysis Based on the Hyderabad Indian Migration Study
Source: PLoS One. 2015 Oct 20;10(10):e0140787. doi: 10.1371/journal.pone.0140787 (PMC4618924; doi:10.1371/journal.pone.0140787)
Supplement: S1 Table — (DOCX) [file pone.0140787.s001.docx]

S1 Table. Bone outcomes and exposures in sibpair design (N=185) with beta coefficient, SEM and p-value.

Coef; unstardized b coefficient, SEM; standard error of mean

^1^linear trend
